# Evidence for Efficacy of Cefiderocol against OXA-48-Containing Isolates from the APEKS-NP and CREDIBLE-CR Trials

**DOI:** 10.1128/aac.01100-22

**Published:** 2022-09-12

**Authors:** Christopher Longshaw, Echols Roger, Anne Santerre Henriksen, Takamichi Baba, Sean Nguyen, Yoshinori Yamano

**Affiliations:** a Shionogi B.V., London, United Kingdom; b Infectious Disease Drug Development Consulting LLC, Easton, Connecticut, USA; c Maxel Consulting ApS, Jyllinge, Denmark; d Shionogi & Co., Ltd., Osaka, Japan; e Shionogi, Inc., Florham Park, New Jersey, USA

**Keywords:** CRE, *Enterobacterales*, OXA-48, beta-lactamases, cefiderocol

## LETTER

We were very interested to read the minireview by Boyd et al. (1) on the classification, identification, epidemiology, and treatment of OXA-48-like carbapenemase-producing *Enterobacterales*. In their discussion of cefiderocol, the authors were correct to point out “it is unclear how many patients had OXA-48-like producers” with regard to the CREDIBLE-CR study (2). Aside from a single poster describing the outcomes of specific carbapenemases from the CREDIBLE-CR study (3), we have not published data specific to clinical efficacy in OXA-48-producing infections except for two cases of bacteremia (4). The authors also point out that registrational clinical trials that usually exclude patients with known carbapenem-resistant infections due to the inactivity of the control antibiotics provide limited high-quality information pertaining to molecularly characterized clinical data on OXA-48- and OXA-48-like pathogens.

Shionogi reviewed both the CREDIBLE-CR and APEKS-NP (5) clinical trial databases, where all pathogens were molecularly characterized. A total of 10 patients with OXA-48-positive *Enterobacterales* who received cefiderocol were identified (Table 1). Seven were from CREDIBLE-CR, and three were from APEKS-NP. All characterized pathogens were Klebsiella pneumoniae species. Of note, three contained OXA-48 along with NDM-1 carbapenemase, and all contained other extended-spectrum (ESBL) and original-spectrum (OSBL) type beta-lactamases. All but two isolates had high-level resistance to meropenem (MIC > 8 μg/mL). All patients but one received cefiderocol as monotherapy, 7 of 10 (70%) patients were judged to have clinical cure at the test-of-cure assessment (cefiderocol MIC range, 0.12 to 4 mg/L), and all survived through day 28.

**TABLE 1:** Summary of CREDIBLE-CR and APEKS-NP database results for Klebsiella pneumoniae

Clinical study	Cefiderocol MIC (μg/mL)	Meropenem MIC (μg/mL)	Site of infection^*a*^	Country	Clinical outcome at TOC	Beta-lactamase profile^*b*^
CREDIBLE-CR	2	4	BSI/sepsis	Spain	Clinical failure	CTX-M-15; SHV-OSBL; TEM-OSBL; **OXA-48**
	4	>64	cUTI	Turkey	Clinical cure	CTX-M-15; SHV-OSBL; TEM-OSBL; **OXA-48; NDM-1**
	0.12	32	cUTI	Turkey	Clinical cure	CTX-M-9-group; SHV-OSBL; TEM-OSBL; **OXA-48**
	1	16	cUTI	Turkey	Clinical cure	CTX-M-15; SHV-OSBL; TEM-OSBL; **OXA-232**
	1	8	cUTI	Turkey	Clinical cure	CTX-M-15; SHV-OSBL; TEM-OSBL; **OXA-48**
	4	>64	cUTI	Korea	Indeterminate	CTX-M-55; SHV-OSBL; **OXA-232; NDM-1**
	1	>64	BSI/sepsis	Thailand	Clinical cure	CTX-M-15; SHV-OSBL; TEM-OSBL; **OXA-232; NDM-1**
APEKS-NP	2	64	RTI	Ukraine	Clinical cure	CTX-M-1-TYPE; SHV-OSBL; TEM-OSBL; **OXA-48**
	1	64	RTI	Russia	Clinical cure	CTX-M-15; SHV-OSBL; **OXA-48**
	0.5	32	RTI	Georgia	Indeterminate	CTX-M-15; SHV-OSBL; TEM-OSBL; **OXA-48**

a BSI, bloodstream infection; cUTI, complicated urinary tract infection; RTI, respiratory tract infection.

b Beta-lactamases in bold type are carbapenemases.

Although these numbers are small, the clinical and microbiological outcomes were consistent with the studies’ overall findings of efficacy against *Enterobacterales*. Together with the demonstrated *in vitro* stability of cefiderocol to OXA-48 hydrolysis (6) and the *in vitro* activity against OXA-48 producers (7–9), we are confident of the potential benefit of cefiderocol treatment for OXA-48-producing *Enterobacterales*.
